# Physiological effects of regular CrossFit^®^ training and the impact of the COVID-19 pandemic—A systematic review

**DOI:** 10.3389/fphys.2023.1146718

**Published:** 2023-04-05

**Authors:** Nicole Meier, Jennifer Schlie, Annette Schmidt

**Affiliations:** Fakultät für Humanwissenschaften, Institut für Sportwissenschaft, Universität der Bundeswehr München, Neubiberg, Germany

**Keywords:** high-intensity functional training, functional fitness training, training behavior, exercise intensity, physical performance, SARS-CoV-2 virus, physiology, lockdown

## Abstract

CrossFit^®^ is a functional fitness training program known for its day-to-day varying “Workouts of the Day” (WOD). In accordance with the ‘CrossFit^®^ Level 1 Training Guide’, regular CrossFit® training sessions consist of Warm-up, Mobility, Skill/Power training, WOD, and Cool-down. Despite the fast-growing and widespread popularity, data on the practical implementation of the training program based on scientific evidence are rare. Therefore, the purpose of this study is to systematically review the existing literature on the physiological effects of regular CrossFit^®^ training in full extent instead of stand-alone WODs and to examine the impact of the COVID-19 pandemic on the training behavior of CrossFit^®^ athletes. A systematic search was conducted following the PRISMA guidelines in April 2022 and updated in July 2022 using the following databases: PubMed, SPORTDiscus, Scopus, and Web of Science. Using the keyword “CrossFit”, 1,264 records were found. Based on the eligibility criteria, 12 studies are included and separated by topics: acute-short term physiological response (n = 8), and impact of the COVID-19 pandemic (n = 4). The results show that studies of regular training sessions were rarely conducted and contradicted the existing knowledge of the physiological demands [e.g., heart rate (HR)] of CrossFit^®^. In detail, included studies demonstrate that training sessions last 30–60 min and provide a progressive increase in cardiovascular load up to maximal effort activity (>90% HR_max_), differing from stand-alone WODs exclusively at high-intensity. Also, scarce research exists on COVID-19-pandemic-induced effects on training behavior, and studies are of moderate to low quality. There is still a lack of comprehensive analyses on the acute physiological effects of regular training sessions and the consequences of the COVID-19 pandemic in the scientific literature. Moreover, the inconsistent terminology used in CrossFit^®^ research complicates generalized conclusions. Therefore, future research on the training methodology of CrossFit^®^ needs to overcome terminological inequalities and examine scientifically the implementation of the concept by considering regular training sessions under practical settings.

## 1 Introduction

CrossFit^®^ is one of the fastest-growing training concepts with over 15,000 affiliated training centers and 5 million athletes (CrossFit, 2022). Driven mainly by the company (CrossFit^®^ Inc. LLC) the new trend sport quickly extended worldwide. The training program involves constantly varied functional movements performed at high-intensity and includes exercises from the main elements of gymnastics (e.g., pull-ups, push-ups, and burpees), weightlifting (e.g., powerlifting, and Olympic weightlifting), and cardiovascular activities (e.g., running, rowing, and jumping) (Glassman, 2004). Here, the day-to-day varying high-intensity workouts are usually referred to as “Workout of the Day” (WOD). Commonly, the workouts are performed quickly, repetitively, and with little or no recovery time between sets. The WODs are designed to perform the required task as fast as possible, namely, “for time” (FT), or to perform the maximum number of repetitions or rounds in a set time interval, namely, “as many rounds as possible” (AMRAP) (Glassman, 2010). To monitor individual performance development by comparing the performance values (e.g., number of repetitions or time to completion) over time or with other athletes, specific Benchmark WODs are provided. These WODs are standardized and performed at irregular intervals, however, each time under the same conditions. The Benchmark WODs include “Girl-WODs” (mostly short and intense workouts, e.g., ‘Cindy’, ‘Fran’ or ‘Helen’), and “Hero-WODs” (often long and hard to complete workouts, e.g., ‘Murph’) (Glassman, 2003).

According to the official ‘CrossFit^®^ Level 1 Training Guide’, regular training sessions at affiliated training centers last about an hour (approximal 45–90 min) and include Warm-up and Mobility exercises, Skill training, in part combined with Power training, followed by the WOD and Cool-down segment with stretching exercises as required (Glassman, 2010). The fast-growing popularity of the training program led also to an increased scientific interest over the last 10 years (Feito et al., 2018a). However, besides CrossFit^®^ training, there exist also other training approaches that focus on functional movements at high-intensity. Thereby, various terms are used in science and practice for this and related types of training, including CrossFit^®^ training, High-intensity multimodal training (HIMT), Extreme conditioning program (ECP), Functional fitness, High-intensity functional training (HIFT) and Mixed modal training (Bergeron et al., 2011; Feito et al., 2018a; Marchini et al., 2019; Dominski et al., 2022; Sharp et al., 2022). However, due to the different terminology used to describe the research on CrossFit^®^ and related training principles, a problem arises. The several terms are unfortunately not used consistently to describe the same type of training method in each case. For this reason, the authors Dominski et al. (2022) summarize the differences between the terms and propose a preferred terminology to refer research on training programs in line with the CrossFit^®^ principles in a consistent manner, namely, Functional fitness training (FFT) (Dominski et al., 2022). The term FFT is therefore intended to comprehensively describe the wide range of training routines that athletes apply to develop their skills in a variety of movement patterns, activities, and energy systems. Nevertheless, to date, different training routines are subsumed under the same term “CrossFit^®^” in the scientific literature, although they differ considerably from each other. In this way, CrossFit^®^ research often examined only the WODs in isolation, instead of regular training sessions in full extent. It follows that studies on the physiological responses (blood lactate concentration [BLC], blood glucose concentration [BGC], blood pressure, heart rate [HR], Oxygen uptake [VO_2_], and Rating of perceived exertion [RPE]) of CrossFit^®^ training exhibit large methodological discrepancies with the recommendations of the official ‘CrossFit^®^ Level 1 Training Guide’ (Glassman, 2010).

By reviewing the current literature, it was observed that most of the studies characterized the responses of stand-alone WODs with duration lengths of less than 10 min (Fernández et al., 2015; Kliszczewicz et al., 2017; Maté-Muñoz et al., 2017; Tibana et al., 2018b; Kliszczewicz et al., 2018; Maté-Muñoz et al., 2018; Mangine et al., 2019; Timón et al., 2019; Kliszczewicz et al., 2021), between 10 and 19 min (Shaw et al., 2015; Escobar et al., 2017; Kliszczewicz et al., 2017; Maté-Muñoz et al., 2017; Tibana et al., 2018b; Durkalec-Michalski et al., 2018; Kliszczewicz et al., 2018; Feito et al., 2019; Mangine et al., 2019; Kliszczewicz et al., 2021; Meier et al., 2021; Toledo et al., 2021), and over 20 min time (Fernández et al., 2015; Maté-Muñoz et al., 2017). A few systematic reviews showed that CrossFit^®^ WODs contain fairly homogenous anaerobic and aerobic characteristics, resulting in substantial metabolite accumulation (e.g., 6–18 mmol/L BLC), and increased markers of muscle damage (Creatine-phosphokinase [CPK], interleukin-6 [IL-6], and IL-10), and muscle fatigue (measured by decreased countermovement jump [CMJ] values, mean power output [MPO], and plank time) (Claudino et al., 2018; Jacob et al., 2020; De Souza et al., 2021). The studies consistently reported that athletes exhibited high BLC either immediately after exercise (Fernández et al., 2015; Shaw et al., 2015; Escobar et al., 2017; Maté-Muñoz et al., 2017; Tibana et al., 2018b; Kliszczewicz et al., 2018; Maté-Muñoz et al., 2018; Feito et al., 2019; Timón et al., 2019; Toledo et al., 2021) or time-delayed after ultra-short workouts <2 min (Meier et al., 2021). Moreover, the mean HR values recorded during the WODs were consistently high (on average 170–180 bpm) (Fernández et al., 2015; Tibana et al., 2018b; Maté-Muñoz et al., 2018; Toledo et al., 2021) and reached values above 90% of the maximum HR (HR_max_) within a brief amount of time (Tibana et al., 2018b), regardless of modalities (AMRAP or FT) (Toledo et al., 2021) or the duration of the workouts (Tibana et al., 2018b). Accordingly, many previous approaches measured the physiological effects of CrossFit^®^ training in the laboratory or isolated clinical testing of specific WODs, instead of how affiliated training centers implement the CrossFit^®^ training program in practice. Thus, there is a research gap on what physiological parameters occur during training sessions under field settings. However, a detailed understanding of acute, short-term responses of regular training in full extent is crucial for optimal adaptation, recovery, and performance, which allows training plans and optimal training interventions to be determined (Schlegel, 2020).

In this regard, substantial changes in training habits deserve attention. So, through the massive consequences of the COVID-19 pandemic, thousands of CrossFit^®^ athletes were forced to adapt their training behavior to the restrictions resulting from the combat against the spread of the SARS-CoV-2 virus since the end of 2019. The governments of several countries undertake early preventive interventions to cope with the global pandemic, e.g., lockdown of cities (Lau et al., 2020), travel warnings and cancellations (Lee et al., 2020), social distancing regulations (Qian and Jiang, 2020; Thunström et al., 2020), and the closure of schools and all non-essential businesses (Song et al., 2021). Specifically, these restrictions extended to the closure of CrossFit^®^ gyms and training facilities, so athletes were unable to perform CrossFit^®^ in their familiar environment and were forced to adapt their training routine to small environments such as a living room or balcony.

To date, no systematic review has examined the acute physiological effects of regular CrossFit^®^ training sessions (contending Warm-up, Mobility, Skill/Power training, WOD, and Cool-down) and characterized the training habits during the COVID-19 pandemic. Thus, the aim of this review is to systematically search the scientific literature on (a) acute, short-term physiological responses of regular CrossFit^®^ training sessions in full extent, and (b) consequences of the COVID-19 pandemic for athletes and the sport of CrossFit^®^, as well as changes in training behavior in response to lockdown and social distancing.

## 2 Methods

### 2.1 Study eligibility

To analyze the findings in the scientific literature, a systematic search was conducted in accordance with the Preferred Reporting Items for Systematic Reviews and Meta-Analyses (PRISMA) guidelines (Liberati et al., 2009; Page et al., 2021). Within this systematic review two research questions (Part A und B) are applied to include research articles in accordance with the objectives. The research question regarding the first aspect adheres to the Population, Intervention, Comparator, Outcomes, Study Design (PICOS) strategy (Amir-Behghadami and Janati, 2020), and regarding the second aspect to the Population, Intertest, and Context (PICo) strategy to determine relevant studies to include (Stern et al., 2014). In both cases, the population includes healthy, adult participants of any gender (≥18 years), and studies on diseases-state participants (e.g., overweight) are not considered. For Part A, the experimental trials must measure at minimum one physiological parameter in acute response to a CrossFit^®^ training (throughout or immediately after the workout), and non-CrossFit^®^-specific HIFT- or HIMT-interventions are not included. In particular, this review only considers studies with a study design that examined the practical implementation of the CrossFit^®^ training concept in accordance with the ‘CrossFit^®^ Level 1 Training Guide’ and consisting of different segments including Warm-up, Mobility, Skill/Power training, WOD, and Cool-down, lasting 30–60 min per training session (Glassman, 2010). Studies that examined the physiological responses of stand-alone WODs are not included. In disputed or unclear cases, a CrossFit^®^ Level 2 trainer (AS) was consulted to assess the compliance of the training intervention with the CrossFit^®^ principles. Further, studies controlled by baseline or preliminary measures of the outcomes (measurements of short-term, acute physiological parameters) are considered. For Part B, the focus of interest is on COVID-19-pandemic-induced changes of the training behavior of CrossFit^®^ athletes in the context of the COVID-19 pandemic and lockdown period. Moreover, only peer-reviewed research studies and original research on humans written in English are eligible. The exclusion criteria are as follows: specific populations (children, seniors, people with disabilities); specific medical or nutrition interventions; non-CrossFit^®^-specific relation; duplicate articles, and not written in English. The detailed inclusion and exclusion criteria according to the PICOS and PICo strategies are presented in Table 1. Articles that include systematic reviews, case reports or series, conference abstracts, dissertations, theses, and book chapters are not considered.

**TABLE 1:** Description of the inclusion and exclusion criteria according to the PICOS and PICo strategies (Stern et al., 2014; Amir-Behghadami and Janati, 2020).

Part - A	Inclusion	Exclusion	Part—B	Inclusion	Exclusion
**P**opulation	Healthy, adult participants (≥18 years)	Diseases-state participants	**P**opulation	Healthy, adult participants (≥18 years)	Diseases-state participants
**I**ntervention	Regular CrossFit^*®*^ training in accordance with the official ‘CrossFit^®^ Level 1 Training Guide’; experimental data	Non-CrossFit^®^-specific workouts; only HIFT/HIMT or stand-alone WOD interventions; questionnaire data	**I**nterest	COVID-19-pandemic-induced changes of the training behavior of CrossFit^*®*^ athletes	Non-CrossFit^®^-specific relation
**C**omparator	Controlled by baseline or pre-intervention measures	Specific medical or nutrition intervention	**Co**ntext	Closure of training facilities, lockdown, and social distancing during the COVID-19 pandemic	Non-COVID-19-specific relation
**O**utcomes	Short-term physiological parameters	Long-term physiological parameters (>48 h)			
**S**tudy design	Cross-sectional, randomized, and non-randomized	Case reports, reviews, and meta-analysis			

**Abbreviations:** PICOS, Population, Intervention, Comparator, Outcomes, Study Design; PICo, Population, Interest, Context.

### 2.2 Search strategy

**FIGURE 1:**
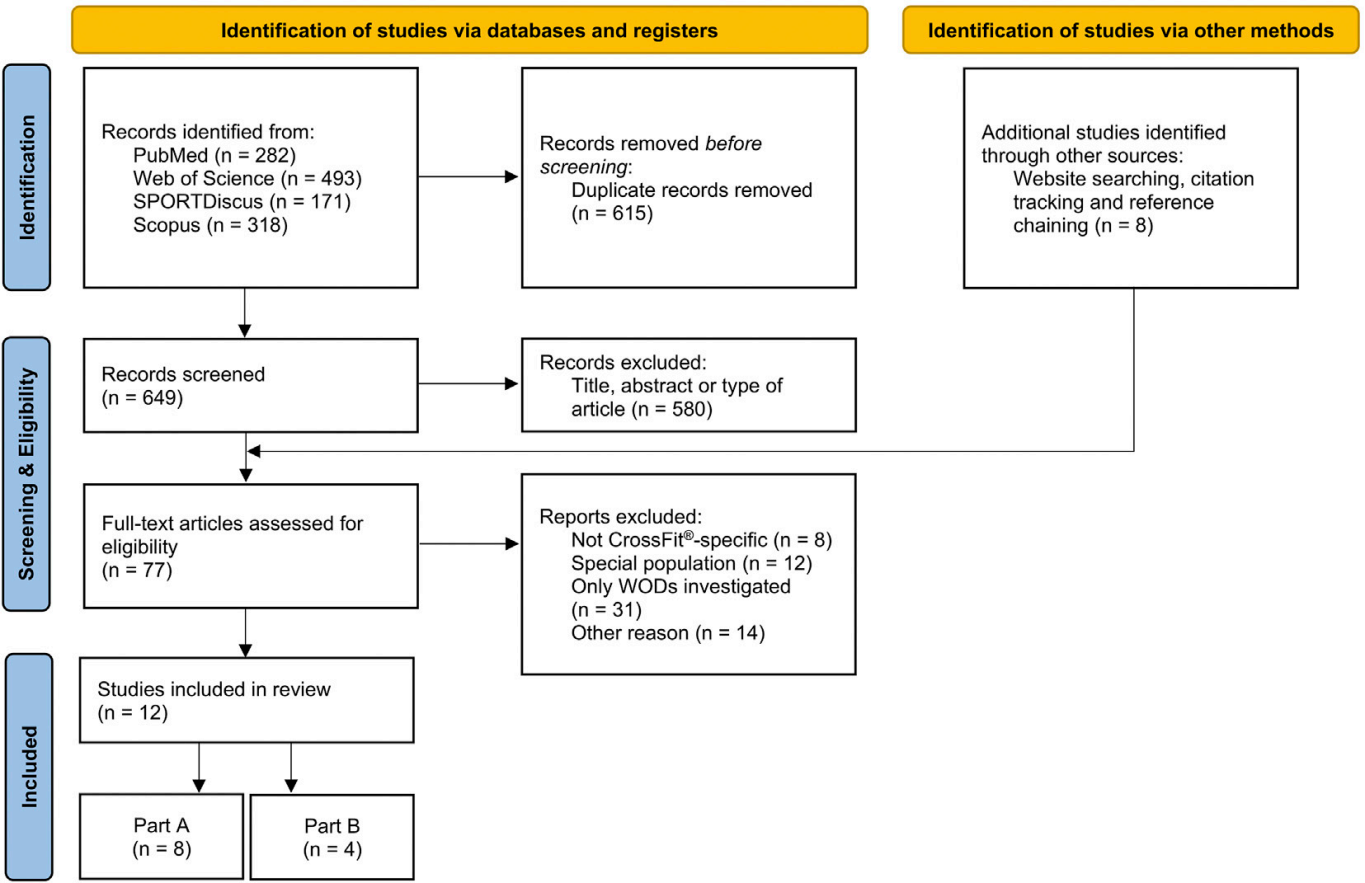
PRISMA flow diagram of the search strategy. Abbreviation: Workout of the Day (WOD).

The electronic literature search was performed in April 2022 and updated in July 2022 using the following databases: PubMed, SPORTDiscus, Scopus, and Web of Science. To identify relevant articles, the search term “CrossFit” was applied without further restriction in order to obtain the maximum number of results. Search results were not limited to any particular number of years. To ensure that relevant articles are included, additional articles were identified through website searching, citation tracking, and reference chaining of relevant original and review articles, see Figure 1.

After the removal of duplicates, two independent researchers (JS and NM) assessed the eligibility of the articles by screening the title and abstract of each record regarding the inclusion and exclusion criteria. In the second phase, the articles were retrieved in full-text read and selected for inclusion in this systematic review by the same two researchers (JS and NM) according to the eligibility criteria. In the case of disagreement regarding the consideration of articles, the opinion of a third reviewer (AS) was requested to resolve the differences.

### 2.3 Data items and collection process

Data extraction was performed by two researchers (JS and NM), subsequently cross-checked, and verified by a third (AS) to avoid errors and reporting bias. Information on the author and year of publication, study design, participants’ characteristics, sample size, data collection, CrossFit^®^ training protocols, and main conclusions were extracted using standardized spreadsheets. For Part A, the measured physiological parameters, and for Part B main outcomes were collected, see Table 2; Table 3. For the physiological parameters, the outcome measures pre-, baseline, and post-intervention (mean ± standard deviation) and the effect size were extracted.

**TABLE 2:** Part A: Acute, Short-term Physiological Responses of CrossFit® training.

References (year)	Sample (n); Profile experience time	Age (years)	Protocol of CrossFit® training; Accordance	Data collection	Main findings	
Dias et al. (2022)	Males (n = 15); ≥ 6 months of CrossFit^®^ experience	26.0 ± 6.5	7 CrossFit^®^ training sessions (60 min) consisting of mobility, warm-up, skill, and WOD segment; according to the CrossFit^®^ training programming template	Pre, (during) and Post	Mobility:	HR_avg_ (% HR_max_) = 49.2 ± 6.5; RPE = 2.7 ± 0.6
					Warm-up:	HR_avg_ (% HR_max_) = 63.1 ± 8.5; RPE = 5.6 ± 0.9
					Skill:	HR_avg_ (% HR_max_) = 67.1 ± 7.1; RPE = 7.3 ± 1.1
					WOD:	HR_avg_ (% HR_max_) = 81 ± 5.8; RPE = 9.8 ± 0.4
					Total:	HR_avg_ (% HR_max_) = 65.1 ± 5.4; RPE = 6.4 ± 0.5
Meier et al. (2022a)	Participants (male = 18; female = 9); beginner (B) ≤ 6 months (n = 8) and experienced (E) > 6 months CrossFit^®^ experience (n = 19)	30.9 ± 4.2	4 CrossFit^®^ training sessions (60 min) consisting of warm-up and movement demonstrations (WU-part), skill and power training (A-part), and WOD (B-part); sessions in an affiliated training center	Pre, (during) and post	Warm-up:	HR_avg_ (% HR_max_) = 57.25 ± 7.5 (B); 59.97 ± 7.3 (E) ↔
					A-part:	HR_avg_ (% HR_max_) = 61.78 ± 9.1 (B); 65.37 ± 7.6 (E) ↔
					B-part:	HR_avg_ (% HR_max_) = 86.92 ± 5.3 (B); 87.77 ± 4.5 (E) ↔
					Total:	HR_avg_ (% HR_max_) = 67.84 ± 6.2 (B); 70.00 ± 5.1 (E) ↔ TL/h = 147.8 ± 28.6 (B); 157.1 ± 24.4 (E) ↔
Garcia-Fernandez et al. (2021)	Participants (male = 24; female = 4); > 18 months of strength training experience	28.7 ± 6.4	Single FFT session (general warm-up, active mobility, specific FFT-warm up, and FFT-workout); guided by a professional qualified in sports science, and exercise descriptions from the 2021 iF3 movements standards	Pre, post, post′4, post′10, and post′20	HR	↑ postexercise (Post: 181.81 ± 8.2 bpm; Avg: 171.52 ± 9.8 bpm)
					BLC	↑ postexercise (Post: 15.23 ± 3.6 mmol/l)
					RPE	↑ postexercise (Post: 15.67 ± 2.0)
					CMJ	↓ Reductions in mechanical variables decreased at post′4, post′10, and post′20
Carreker et al. (2020)	Males (n = 11); ≥ 6 months of CrossFit^®^ experience	27.2 ± 3.3	CrossFit^®^ ‘Murph’-WOD (approx. 45 min); “Hero”-WOD according to official CrossFit^®^ website	Pre and post	HR	↑ postexercise (Peak: 185.63 ± 7.6 bpm: Avg: 168.81 ± 6.4 bpm)
					BLC	↑ postexercise (Post: 10.01 ± 3.0 mmol/l; Change: 7.60 ± 3.50 mmol/l)
Cavedon et al. (2020)	Males (Higher Training, HT = 13; Lower Training, LT = 11); ≥ 1 year of CrossFit^®^ training experience	28.2 ± 3.4	Specific CrossFit^®^-warm-up followed by ‘Fran’-WOD; “Hero”-WOD according to official CrossFit^®^ website, and supervised and scored by a certified CrossFit^®^ Level 1 trainer	Pre, post, and post′15	HR_avg_	LT (% HR_max_) = 94.1 ± 3.7; HT = 92.7 ± 5.3 ↔
					HR_peak_	LT (% HR_max_) = 98.3 ± 3.7; HT = 97.4 ± 5.3 ↔
					BLC	Pre (LT;HT): 2.0 ± 1.0; 2.0 ± 0.9 mmol/l ↔ Post (LT;HT): 14.6 ± 2.4; 14.8 ± 2.3 mmol/l ↔ Post′15 (LT;HT): 12.8 ± 2.0; 13.8 ± 2.2 mmol/l ↔
					BGC	Pre (LT;HT): 74.3 ± 17.0; 69.4 ± 13.8 mg/dl ↔ Post (LT;HT): 97.4 ± 27.1; 90.8 ± 31.1 mg/dl ↔ Post′15 (LT;HT): 108.8 ± 24.1; 97.9 ± 23.4 mg/dl ↔
Faelli et al. (2020)	Males (CrossFit^®^ = 10; resistance training, RT = 10); > 1 year of experience in CrossFit^®^ or resistance Training	CrossFit^®^ group 24.6 ± 3.4 (RT group 26.3 ± 3.6)	24 CrossFit^®^ training sessions (60 min) consisting of Warm-up and mobility, WOD, and Cool-Down; sessions in an affiliated training center and supervised by a certified CrossFit^®^ Level 1 trainer	Pre and post′30	Cortisol	↑ Pre: 6.14 ± 0.7; Post′30: 19.94 ± 0.9 μg/dl (RT: ↓)
					IL-1ß	↓ Pre: 17.04 ± 0.2; Post′30: 7.94 ± 0.3 pg/ml (RT: ↓)
					Uric acid	↑ Pre: 8.68 ± 0.6; Post′30: 11.62 ± 0.4 mg/dl (RT: ↑)
Cronin et al. (2016)	Participants (male = 30; female = 20); well-trained	Males 30.7 ± 9.9; females 29.5 ± 8.3	3 CrossFit^®^ training sessions (30-47 min); sessions in two affiliated training centers	Pre and post	Sweat loss	↑ in men vs. women (Men: 0.894 ± 03 l; women: 0.525 ± 0.2 l)
					Rate	↑ in men vs. women (Men: 1.663 ± 0.5 l/h; women: 0.886 ± 0.3 l/h)
					%BM	↑ in men vs. women (Men: 0.99 ± 0.3%; women: 0.78 ± 0.2%)
					Fluid intake	↔ Men: 0.592 ± 0.2 l; women: 0.565 ± 0.2 l
					Fluid replacement	↑ in women vs. men (Men: 75.1 ± 46.8%; women: 127.8 ± 82.1%)
Tibana et al. (2016)	Men (n = 9); trained > 6 months	26.7 ± 6.6	2 training sessions consisting of strength and power exercises, gymnastic movements, and metabolic conditioning (AMRAP-workout); members of extreme conditioning program community	Pre, post, and 24-h and 48-h post	BLC	↑ (Session 1: 1.20 ± 0.41 to 11.84 ± 1.34; Session 2: 0.94 ± 0.34 to 9.05 ± 2.56 mmol/l)
					BGC	↑ (Session 1: 81.59 ± 10.27 to 114.99 ± 12.52; Session 2: 69.47 ± 6.97 to 89.95 ± 19.26 mg/dl)
					IL-6	↑ post-training session 1 and 2
					IL-10	↑ post-training session 1
					Osteoprotegerin	= post-training session 1 and 2

**Abbreviations:** AMRAP, As many rounds as possible ; Avg, Average; HRavg, Average heart rate ; B, Beginner; BGC, Blood glucose concentration; BLC, Blood lactate concentration; BM, Body mass; CMJ, Countermovement jump; FFT, Functional fitness training ; E, Experienced athletes ; HT, Higher-Training group ; IL, Interleukin; iF3, International Functional Fitness Federation ; LT, Lower-Training group; HRmax, Maximal Heart rate ; RPE, Rating of perceived exertion ; RT, Resistance training group ; TL, Training load; WOD, Workout of the Day.

**TABLE 3:** Part B: Consequences of the COVID-19 Pandemic to CrossFit^®^ athletes.

References (year)	Sample (n); age (years)	Measures	Context	Main findings
Meier et al. (2022b)	Participants (male = 290; female = 192; diverse = 2) divided in CFA (n = 266) and WLA (n = 218); 31 (range 18–65)	Online survey to identify changes in training behavior and differences between CrossFit^®^ and weightlifting, with a focus on purchasing habits, body mass changes, and acceptance of digital sports offerings	Restrictions of the nationwide lockdown in **Germany** from mid-March until June 2020	↑ CFA and WLA bought new equipment
				↑ Usage of digital sport offers increased
				↓ CFA subgroup (n = 142) showed a weight loss of at least 5 kg
Araujo et al. (2022)	Females (n = 197); 32	Online survey about frequency, duration, and intensity of training and data of CrossFit^®^ athletes related to the COVID-19 pandemic with focus on the prevalence of urinary incontinence (UI) before and during the quarantine	Quarantine and closure of non-essential services (incl. Affiliated training centers) in **Brazil** starting March 2020	↑ Body weight exercises were most performed
				↓ Decrease in training intensity (of 64% of participants)
				↓ Appearance of UI decreased (from 32% to 14%))
Cataldi et al. (2021)	Participants (male = 18; female = 12); 18.26 ± 0.52	Study to examine the effectiveness of a CrossFit^®^ program in mitigating fitness deficits caused by COVID-19 prevention interventions, with an intervention group (IG) that completed an 8-week CrossFit^®^ training program and a control group (CG)	Social distancing and closure of sport centers in **Italy** in the summer of 2020	↑ Significant improvements for all fitness tests in the IG
				↑ Higher scores for the Regulatory Emotional Self-Efficacy scale (RESE) negative and positive scales in the IG
				= no significant difference for the fitness tests in the CG (except for the push-up test)
Redwood-Brown et al. (2021)	Participants (male = 799; female = 1006); range 18–65	Online survey addressing self-reported training history, health and lifestyle history, nutritional customs, present training status and suspected levels of exposure to COVID-19 of CrossFit^®^ athletes	Restrictions of the nationwide lockdown in **United Kingdom’s** from May until June 2020	= 45% reported no changed exercise habits
				CrossFit^®^ participation (minutes of exercise) was indicative of a lower BMI and was not shown to impact perceptions of disease, particularly relating to probability of COVID-19 infection

**Abbreviations:** BMI, Body mass index ; CG, Control group; CFA, CrossFit athletes ; IG, Intervention group ; RESE, Regulatory Emotional Self-Efficacy scale ; UI, Urinary incontinence ; WLA, weightlifting athletes.

### 2.4 Assessment of risk of bias

Cochrane Collaboration’s risk of bias assessment tool (RoB 2) is applied to evaluate the risks of bias in each included study (Sterne et al., 2019). Therefore the authors (JS and NM) evaluated selection bias (random sequence generation and allocation concealment), performance bias (blinding of participants and researchers), detection bias (blinding of outcome assessment), attrition bias (incomplete outcome data), reporting bias (selective reporting), and other biases (anything else) by rating the risk of bias as low, some concerns, high, or no information. A third researcher (AS) was involved to discuss any disagreements.

## 3 Results

### 3.1 Study search

The first search identified 1,264 titles from the databases. Initially, 615 records containing duplicates were excluded. Subsequently, after reviewing the titles and abstracts of 649 articles, 77 articles (11.9%) went through a full-text review to assess eligibility. In addition, 8 articles were identified from reference lists and article chaining, and the full-texts were also screened. A total of 12 articles meet the inclusion criteria, including 8 studies on physiological effects (Part A), and 4 studies on COVID-19-pandemic-induced changes of the training behavior (Part B). All articles were published between 2016 and 2022 and written in English.

### 3.2 Part A: Acute, short-term physiological responses

The included studies of Part A (*n* = 8) of this systematic review are most of a cross-sectional design. Here, physiological responses to CrossFit^®^ training are described in terms of pre- and post-training (*n* = 5) (Cronin et al., 2016; Tibana et al., 2016; Carreker and Grosicki, 2020; García-Fernández et al., 2021; Dias et al., 2022) and/or compared between groups (*n* = 3) (Cavedon et al., 2020; Faelli et al., 2020; Meier et al., 2022b). Study sample sizes range from 9 to 50 participants, with more than half of the studies including only men (*n* = 5) (Tibana et al., 2016; Carreker and Grosicki, 2020; Cavedon et al., 2020; Faelli et al., 2020; Dias et al., 2022). Among the participants, the inclusion criteria of four studies are at least 6 months of CrossFit^®^ experience (Tibana et al., 2016; Carreker and Grosicki, 2020; Meier et al., 2022b; Dias et al., 2022), two studies at least 12 months (Cavedon et al., 2020; Faelli et al., 2020), and one study at least 18 months (García-Fernández et al., 2021); however, in one study the training status “well-trained” is not further specified (Cronin et al., 2016). Adherence to the CrossFit^®^ training principles of the interventions is present across the majority through training at an affiliated training center and supervision by CrossFit^®^ Level 1 or Level 2 trainer (Cronin et al., 2016; Cavedon et al., 2020; Faelli et al., 2020; Meier et al., 2022b). In addition, interventions are implemented according to the CrossFit^®^ training programming template (Carreker and Grosicki, 2020; Dias et al., 2022) or movements standards set by the International Functional Fitness Federation (iF3) (García-Fernández et al., 2021), and subjects are selected from the ECP community (Tibana et al., 2016). In accordance with the inclusion criteria of this review, the length of training protocols range from 30 to 60 min. Thereby, multiple training sessions in full extent consistent with the official ‘CrossFit^®^ Level 1 Training Guide’ are found. Four studies analyzed standard 1-h training sessions that typically consisted of Warm-up and Mobility exercises, Skill/Power training, the WOD, and Cool-down segments in this or a similar setup (Cronin et al., 2016; Faelli et al., 2020; Meier et al., 2022a; Dias et al., 2022). Another four investigated training sessions of CrossFit^®^-specific Warm-up, followed by AMRAP- or FT-workouts with strength and power exercises, gymnastic movements, and metabolic conditioning (Tibana et al., 2016; García-Fernández et al., 2021), or “Hero”-WODs, namely, ‘Fran’ consisting of 21–15–9 repetitions thrusters (95/65 lb) and pull-ups (Cavedon et al., 2020) and ‘Murph’ consisting of 1-mile run, 100 pull-ups, 200 push-ups, 300 air squats, 1-mile run (Carreker and Grosicki, 2020). These sessions lasted non-etheless up to 30–47 min.

As an acute response, the investigation analyzed several physiological parameters. The variables analyzed are HR, BLC, BGC, RPE, CMJ, Cortisol, IL-1ß, IL-6, IL-10, Sweat loss and rate, Fluid intake and replacement, Uric acid, and Osteoprotegerin. In five included studies, HR was measured pre-, during-, and post-workout (Carreker and Grosicki, 2020; Cavedon et al., 2020; García-Fernández et al., 2021; Meier et al., 2022b; Dias et al., 2022). When HR was specified separately for each segment of CrossFit^®^ training, a progressive cardiovascular load increase was described during 1-h training sessions (Meier et al., 2022b; Dias et al., 2022). As a result, mean HR and RPE increased significantly during each segment, and beginner and experienced athletes were able to exercise to maximal capacity with no significant differences in cardiovascular responses to the training stimulus. Vigorous intensity activity (<85% of HR_max_) was achieved initially during the WOD segment. However, the mean HR of the total training sessions remained lower, ranging between 168.81 ± 6.4 bpm (Carreker and Grosicki, 2020) and 171.52 ± 9.8 bpm (García-Fernández et al., 2021), or 65.1% ± 5.4% of HR_max_ (Dias et al., 2022) and 67.84% ± 6.2% of HR_max_ (Meier et al., 2022b). Only one study reported that the mean HR of the total training unit exceeded 90% of HR_max_, both in lower- and higher-trained athletes (Cavedon et al., 2020). In addition, several studies analyzed blood lactate (Tibana et al., 2016; Carreker and Grosicki, 2020; Cavedon et al., 2020; García-Fernández et al., 2021). In agreement, it was found that the BLC increased immediately after the training sessions. The increase in values occurred significantly from pre-to post-exercise and reached 9.05–15.23 mmol/L. Also, a number of studies showed changes in glycemia after exercise. Similar to lactate, the BGC increased from 81.59 ± 10.27 to 114.99 ± 12.52, or from 69.47 ± 6.97 to 89.95 ± 19.26 mg/dL after two different training sessions consisting of strength and power exercises, gymnastic movements, and metabolic conditioning (Tibana et al., 2016). Another study reported a moderate increase from pre-to post-exercise (‘Fran’-WOD) in both groups (lower- or higher-trained athletes, respectively, +30.8% or +31.1%) (Cavedon et al., 2020). Mean sweat loss amounted to 0.746 ± 0.305 L during training sessions lasting 30–47 min, and the sweat rates were nearly two-fold higher in men (1.663 ± 0.478 L/h) compared with women (0.886 ± 0.274 L/h) (Cronin et al., 2016). When examining muscle fatigue over a 20-min period after an FFT workout, one study found that CMJ mechanical variables decreased 4, 10, and 20 min post-exercise (García-Fernández et al., 2021). Further, the analyzed variable of hormonal response was cortisol. As an acute effect, cortisol levels increased after CrossFit^®^ training sessions in contrast to the resistance training group (Faelli et al., 2020). This study also examined the inflammatory responses of the CrossFit^®^ training group compared to the resistance training group. Biomarkers of muscle damage were IL-1ß, IL-6, and IL-10 (Tibana et al., 2016; Faelli et al., 2020). While IL-1ß decreased after both CrossFit^®^ and resistance training (Faelli et al., 2020), IL-6 increased WOD-independently and IL-10 increased as a function of the WOD (Tibana et al., 2016).

### 3.3 Part B: Consequences of the COVID-19 pandemic

Included studies on COVID-19-pandemic-induced effects on the training behavior of CrossFit^®^ athletes are most of cross-sectional design. Three of the four observations use online surveys to examine participants’ training history and their self-reported changes during the pandemic period (Redwood-Brown et al., 2021; Meier et al., 2022a; Araujo et al., 2022). Another investigation aims to examine the effectiveness of a CrossFit^®^ program to mitigate the fitness deficits caused by COVID-19 prevention using a prospective, controlled intervention study of young adolescents (Cataldi et al., 2021). In the studies, sample sizes range from 30 to 1,806 participants (Cataldi et al., 2021; Redwood-Brown et al., 2021; Meier et al., 2022a; Araujo et al., 2022), with one study including only female participants (Araujo et al., 2022). The time span used for the surveys is the first year of the COVID-19 pandemic immediately following the outbreak. From March 2020 to June of the same year, all studies collected their data during the first lockdown. The consequences of the restrictions during the COVID-19 pandemic on the CrossFit^®^ sport are considered at this point from 3 countries in Europe (Germany, Italy, and the United Kingdom) (Cataldi et al., 2021; Redwood-Brown et al., 2021; Meier et al., 2022a) and South America (Brazil) (Araujo et al., 2022). The impact of the first lockdown in Germany on the use of digital sports offers, training habits, body weight changes, and purchase of sports equipment of CrossFit^®^ and weightlifting athletes was considered by Meier et al. (2022a). The data presented show that the athletes were able to continue their training despite the massive restrictions. Both CrossFit^®^ and weightlifting athletes purchase new equipment for a home gym and the use of digital sports increased significantly across all age groups among CrossFit^®^ athletes, in contrast to weightlifters. A comparison during the lockdown even showed that one group of CrossFit^®^ athletes (n = 142) reported a reduction of 5 kg or more of body mass, while the value of the weightlifting athletes remained constant. Another study compared the prevalence of urinary incontinence (UI) before and during the COVID-19 lockdown in Brazil among CrossFit^®^ practicing women and showed a decrease from 32% to 14% during the lockdown period. In addition, the results reveal that most (98.5%) of the participants were able to continue with CrossFit^®^ training and physical exercise routine at home during times of social distancing, even in small environments, such as a balcony. Nevertheless, a decrease in training intensity was noted in 64% of the respondents, as shown by bodyweight exercises, such as air squats (98.2%) were mostly performed. Thus, the authors concluded that the forced reduction of training intensity led to a decrease in the prevalence of urinary incontinence among female athletes (Araujo et al., 2022). Furthermore, the purpose of the study by Redwood-Brown et al. was to establish whether habitual CrossFit^®^ participation is associated with lower body mass index (BMI) values during the United Kingdom’s nationwide lockdown and to further investigate how habitually trained CrossFit^®^ athletes to perceive their COVID-19 susceptibility. The main findings indicated that self-reported CrossFit^®^ participation (measured in minutes of exercise) was indicative of a lower BMI and that athletes did not consider their training history to impact the probability of infection. Furthermore, the results provide insight into the training behaviors of CrossFit^®^ athletes during a period of national lockdown. Over 45% of participants declared that their training habits remained unchanged, and more than 50% reported that their mental wellbeing did not change during this period (Redwood-Brown et al., 2021). To mitigate fitness deficits caused by restrictions during the COVID-19 pandemic period in Italy, an intervention study examined the effectiveness of an 8-week CrossFit^®^ training program. The findings of Cataldi et al. present that CrossFit^®^ training positively affects the general physical fitness and mental attitude measured by the Regulatory Emotional Self-Efficacy scale (RESE) in healthy adolescents compared to a control group (Cataldi et al., 2021).

### 3.4 Risk of bias

**FIGURE 2:**
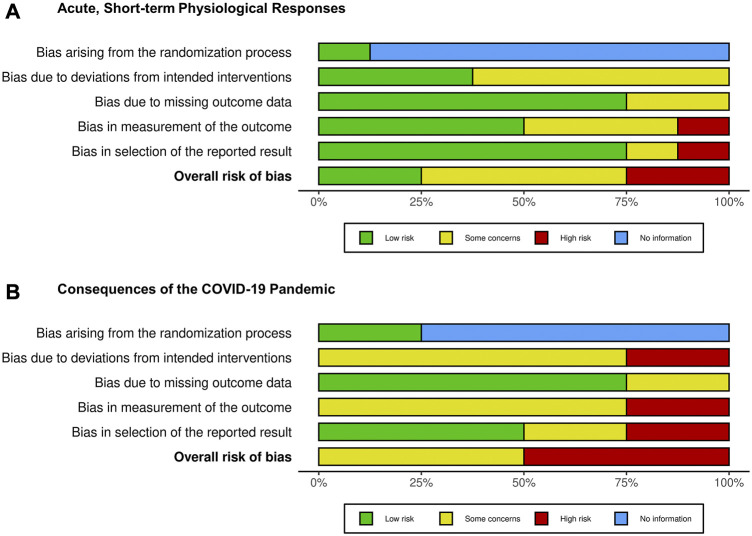
Cochrane risk of bias assessment across the included studies from Part **(A)** and Part **(B)** of the systematic review.

The results of the methodological quality assessment across all included studies are shown in Figure 2 based on the percentage distribution of quality. Overall bias risks in the studies in Part A is low (n = 2; 25.0%), some concerning (n = 4; 50.0%), and high (n = 2; 25.0%). In 7 of 8 studies, the procedures for generating a random sequence and concealing allocation were unclear. In Part B, the studies show the overall bias risk some concerning (n = 2; 50.0%) and high (n = 2; 50.0%). Detailed evaluation of risk of bias of the included studies are presented in Supplementary Figure S1, S2.

## 4 Discussion

### 4.1 Key findings

The purpose of this study is to systematically review the existing literature on the physiological response of regular CrossFit^®^ training conducted in accordance with the ‘CrossFit^®^ Level 1 Training Guide’ and consisting of different segments including Warm-up, Mobility, Skill/Power training, WOD, and Cool-down, lasting 30–60 min per training session. Further, this systematic review aims to examine the impact of the COVID-19 pandemic on the training behavior of CrossFit^®^ athletes. The results showed that studies of CrossFit^®^ training in full extent are rarely conducted and contradict the existing knowledge of the physiological demands about CrossFit^®^ workouts in several ways. Also, scarce research exists on COVID-19-pandemic-induced effects on training behavior and studies are common of moderate to low quality.

**FIGURE 3:**
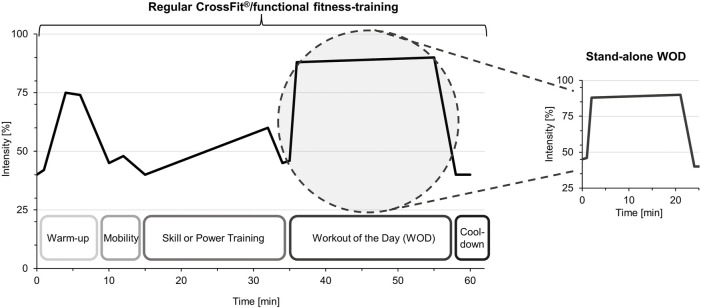
Differentiation between investigations of regular CrossFit^®^/functional fitness-training *versus* stand-alone WODs.

Nevertheless, the different training sessions indicate similar physiological responses, as evidenced by the mean HR at light intensity (65%–70% of HR_max_) and reaching vigorous intensity (<85% of HR_max_) only during the WOD. Specifically, 1-h training sessions provide a progressive increase in cardiovascular load up to maximal effort activity (>90% HR_max_). So, the high intensity that characterizes CrossFit^®^ is mainly associated with the WOD and concerns not a complete 1-h training session in general. Comparing these findings with corresponding literature highlights an important difference. Several previous reviews on the physiological and metabolic response of CrossFit^®^ training revealed the intense nature of CrossFit^®^ with mean HR above 90% of HR_max_, large increases in BLC, and high muscle fatigue immediately following WODs. However, in most of the included studies, only stand-alone WODs are examined separately and not regular training sessions as CrossFit^®^ is usually performed in practice in affiliated training centers. Thus, for the first time, this review presents a summary of the physiological response of the practical application of the CrossFit^®^ training concept. The discrepancy to the previous data resulting from differences in study design (CrossFit^®^ training in full extent vs stand-alone WODs) is outlined as follows. A comparison between shorter and longer CrossFit^®^ workouts, and between training modalities such as AMRAP and FT were previously examined and found that the protocols achieved HR values above 90% HR_max_ without significant differences (Tibana et al., 2018b; Forte et al., 2021; Toledo et al., 2021). In this regard, a study included in this review also confirms this finding based on a mean HR of 94.1 ± 3.7 in lower-trained and 92.7 ± 5.3 of HR_max_ in higher-trained athletes. However, in this study, HR was measured separately during the WOD of the training session, so equal cardiovascular response was detected. Similarly, the studies by Dias et al. and Meier et al. reported the mean HR of the WOD segment of the training session separately as associated with maximal effort activity (Meier et al., 2022b; Dias et al., 2022). As a result, it is important to note that the WOD is only one segment of a training session, see Figure 3. Additional segments, such as technical Skill training or Power training, are crucial for performance progression and developing the ability to perform coordinately demanding movements at high intensity in a health-preserving manner.

However, the main blood marker studied was BLC, which showed a homologous response by high levels, in agreement with the existing literature. Given that there are usually no standard break times in CrossFit^®^ and participants choose the breaks themselves depending on the workout (however, some workouts have set break intervals, e.g., ‘Fight Gone Bad’), the BLC may remain at high levels during the training sessions. Due to the length of the training of up to 60 min, increased values occurs immediately after the end of the exercise and are not time-delayed as another study of ultra-short WOD recently indicated (Meier et al., 2021). Blood glucose was another variable observed. According to similar research, blood glucose levels rise in response to CrossFit^®^ workouts as a result of an increase in catecholamines to supply energy requirements. (Kliszczewicz et al., 2017; Timón et al., 2019). In other studies, however, no significant acute changes in blood glucose levels were observed (Shaw et al., 2015; Perciavalle et al., 2016; Coco et al., 2019). In summary, the BLC, BGC, HR, and RPE values indicate a high exposure to the physiological system. Accordingly, the RPE also follows a progressive increase within a 1-h training session. So, participants were motivated to maximum effort, resulting in the RPE increasing to maximum values at the end of the training session. Considering that RPE is correlated with HR, monitoring of exercise load is enabled as shown previously (Tibana et al., 2018a). Muscle fatigue from CrossFit^®^ training is evidenced by impairment in various CMJ parameters, as observed previously also during and after several stand-alone WODs (Maté-Muñoz et al., 2017; Maté-Muñoz et al., 2018). In addition, muscle fatigue appears to coincide with increased muscle damage and pro-inflammatory markers, as revealed by increased IL-6 and IL-10 levels. The leukocyte-produced IL-6 is considered a multifunctional cytokine that represents an inflammatory response to movement and may also trigger an increase in IL-10 (Suzuki et al., 2020). In contrast, the hormonal response expressed by an increase in cortisol levels might also be explained by psychological factors, as observed in different types of athletes. The demanded goal of performing the required task in as many rounds as possible or in the fastest time potentially affects the psychological stress of CrossFit^®^ training and establishes an environment that promotes an increase in cortisol levels (Mangine et al., 2018). The inflammatory, endocrine, and muscle fatigue responses are consistent with the responses of separate WODs, and thus are likely to be influenced mostly by the high-intensity segment at the end of the training sessions (Kliszczewicz et al., 2017; Maté-Muñoz et al., 2017; Kliszczewicz et al., 2018; Mangine et al., 2018; Timón et al., 2019; Tibana et al., 2022).

In other words, it also depends on the length of the WOD within a 1-h training session, how much inflammation and muscle damage occurs. Consequently, slower recovery from muscle fatigue needs to be considered when planning training on subsequent days to avoid potential injuries. The outcomes indicate that further studies are needed to evaluate the intensity and physiological demands of regular CrossFit^®^ training in full extent to determine safe intervals between training sessions and to guide training planning. That becomes particularly relevant when the training no longer takes place in affiliated centers under the supervision of qualified trainers (CrossFit^®^ Level 1 or Level 2 certified) on-site, as during lockdown and quarantine periods of the COVID-19 pandemic. For this reason, this research also reviews the impact of the COVID-19 pandemic on the exercise behavior of CrossFit^®^ athletes. So, the presented data provide for the first time a detailed insight into how the athletes dealt during the COVID-19 lockdown and quarantine period. Special mention deserves the finding that despite the massive restrictions, the CrossFit^®^ athletes be able to continue their training and, in part, were able to promote health aspects by improving body composition (Meier et al., 2022a), reducing UI (Araujo et al., 2022), and maintaining wellbeing (Redwood-Brown et al., 2021). It is also evident that practicing CrossFit^®^ at an affiliated training center entails more than just fitness training. All included studies suggest that the social environment created in CrossFit^®^ plays a crucial role (Whiteman-Sandland et al., 2016; Heinrich et al., 2017). In this context, the use of online social networks by CrossFit^®^ affiliates might influence the observed results. Thereby, the reasons for the high adherence to CrossFit^®^ training and the associated consequences for public health are of particular interest in the future. However, compared to a control group or other sports disciplines (weightlifting athletes) practicing CrossFit^®^ seems to be a positive relation with more physical activity during the lockdown period (Cataldi et al., 2021; Meier et al., 2022a). In other types of sports, reduced training behavior occurred during the pandemic period, for example, among Spanish basketball players (Lorenzo Calvo et al., 2021), or reduced training time on the ball among Austrian soccer players (Schüttler et al., 2021). Interestingly, the results suggest that the training habits of most CrossFit^®^ athletes remained unchanged during the lockdown period and that online training increased in importance across all age groups in contrast to other sports, for example, in amateur golf (Huth and Billion, 2021). In this way, the benefits of staying physically fit during lockdown or quarantine times should be mentioned. In accordance with the results of Gil et al., showing that physical strength allows a better recovery from a COVID-19 infection (Gil et al., 2021), maintaining CrossFit^®^ training also during challenging times may be recommended. So, being physically active also has substantial effects on emotional wellbeing (McAuley and Rudolph, 1995). Still, data and evidence on the impact of CrossFit^®^ training on mental health during the pandemic times are limited. Since current evidence suggests that several mental health problems are associated with the COVID-19 pandemic (Hossain et al., 2020), future research should also emphasize on psychological aspects of CrossFit^®^, as, for example, recently by authors Dominski et al. in a systematic review, and its potential positive effect on health-related outcomes, especially during challenging times (Dominski et al., 2021).

Taken together, the investigations provide new insights into the physiological parameters of CrossFit^®^ training and changes of training behavior during the COVID-19 pandemic period, offering an initial starting point for prospective, controlled, long-term, intervention studies (Feito et al., 2018b; Brisebois et al., 2018; Cosgrove et al., 2019). So far, several intervention studies showed the applicability also for untrained, overweight, or disabled people and the benefits of CrossFit^®^ training (Patel, 2012; Eather et al., 2016; Dehghanzadeh Suraki et al., 2021). So, the MedXFit-Study examined for the first time the effects of 6-month CrossFit^®^ training on strength, mobility, back health, and wellbeing in sedentary and inactive workers, and scientifically confirms the performance- and health-promoting effects (Brandt et al., 2022). This is consistent with the results of this systematic review in that CrossFit^®^ provides a moderate increase in load within a training session, avoiding overtraining and overexertion due to scalability to the individual fitness level of the participants.

### 4.2 Limitations

However, some limitations are noted in the included studies. Sample sizes are small, often include only male participants, and the age of the participants focus on a range between 20 and 30 years in average. As the physiological responses may differ by gender or age, further studies are needed to verify whether the physiological responses are consistent among women attending CrossFit^®^ training. Due to the differences in results and the small number of studies or sample sizes, however, a number of parameters are inconclusive and thus limit the possibility in drawing generalized conclusions. In addition, data are only available for the first year of the COVID-19 pandemic. There is a lack of data on the following years and the persistence of the observed effects in the post-pandemic period. However, considering these limitations, some practical recommendations may be derived.

### 4.3 Practical application

Despite the limitations, some recommendations for the practical application of the CrossFit^®^ training concept are to be derived. First, it is recommended to monitor the HR of all participants in a guided training session to follow the cardiovascular load increase. In this way, it is possible to ensure that all participants receive the optimal training stimulus without being overwhelmed or not sufficiently exercised. So, coaches and trainers should ensure that all participants, regardless of experience or fitness level, exercise together to their maximum capacity. Also, the first phase of a 1-h training session ought not to be too exhausting (indicated by RPE and % of HR_max_) in terms of muscle glycogen and blood lactate so as not to interfere with the following segments (Schlegel, 2020). Overall, it depends on the right dose of intensity. Therefore, it is necessary to affect the set intensity carefully by the combination of the individual components, which is especially crucial for the first half of the training session. The objective is to reach the maximum intensity in the last segment of the training session during the WOD for optimal performance progress. Also, within a training session, power progression is beneficial to take advantage of the positive effects of high-intensity training without negative interference (Schlegel, 2020). As the present results show, the duration of the WOD within a 1-h training session also determines muscle fatigue and needs to be considered in the scheduling of subsequent training days. In addition, data obtained during the COVID-19 pandemic demonstrate how beneficial maintaining CrossFit^®^ training may be in terms of public health while training centers are closed. Thus, the results recommend sustaining exercise practice as it is respectively possible. When there is inadequate sports equipment and limited space, it is at least feasible to train using bodyweight exercises in a minimum amount of space. In this context, a strong sense of community and social support among the members in the affiliated training centers contributes to motivation and builds adherence to the training concept (Whiteman-Sandland et al., 2016; Dominski et al., 2020; Patterson et al., 2020). Social networking and relationship building may also occur online through digital sports offers (Vuorenlinna, 2021). Future sports offerings are expected to additionally include such services, regardless of lockdown periods.

### 4.4 Perspectives for research

This review highlights important methodological differences in study design (regular CrossFit^®^ training in full extent vs stand-alone WODs) among investigations of the physiological demands of the training program. In this way, the characterization of stand-alone WODs corresponding to HIFT-workouts as high-intensity training is plausible in principle. However, conclusions drawn from consideration of individual WODs should be related to what was studied, namely, stand-alone HIFT-workouts and not CrossFit^®^ training in general. Overall conclusion for CrossFit^®^ training should only made of studies considering regular training habits in accordance with the ‘CrossFit^®^ Level 1 Training Guide’. A single-sided view occurs when the effectiveness of CrossFit^®^ training is primarily based on investigations of the WODs and the other training components are systematically not considered (e.g., Skill/Power training). Moreover, through interference of the study results of separate stand-alone WODs or HIFT-workouts and CrossFit^®^ training in full extent, the conclusions become less precise. In fact, there is a systematic bias in the scientific literature by subsuming standalone WODs and CrossFit^®^ training sessions under the same heading of CrossFit^®^. To date, only 8 investigations provide insights into practical training settings and are rated low to medium in quality assessment. In further work, studies focused on the short- and long-term effects of practical training conditions are needed to evaluate the effectiveness of the CrossFit^®^ training concept and fill this research gap. These studies should ensure that the training protocol is familiar with the recommendations of the official ‘CrossFit^®^ Level 1 Training Guide’ and provide a description of the protocol used. In this way, the terminology of the training methodology applied for the conducted research needs to be more precise. As in previous years, studies in this regard use a variety of terms to describe CrossFit^®^ training practice, including CrossFit^®^, HIFT, HIMT, FFT, ECP, and Mixed modal training (Bergeron et al., 2011; Feito et al., 2018a; Marchini et al., 2019; Dominski et al., 2022; Sharp et al., 2022). Also, several excluded records of this review use the term CrossFit^®^ for analysis of only separated workout instead of regular training session. The inconsistency of the terminology extends so far that even in systematic reviews, the term CrossFit^®^ training is used to summarize the physiological effects of isolated HIFT-workouts and as far as CrossFit^®^ training session in full extent without further differentiation (Feito et al., 2018a; Claudino et al., 2018; Jacob et al., 2020; Schlegel, 2020; De Souza et al., 2021). This fact complicates attempts to derive evidence-based recommendations for this type of training program. For this reason, this review indicates the appropriate use of the term CrossFit^®^ training by including only studies of training sessions in full extent under practical settings. In this manner, the opinion is supported that a separate analysis of stand-alone WODs that includes solely metabolic conditioning training at high-intensity does not comprehensively reflect the training methodology of CrossFit^®^ and are insufficient to adequately characterize and understand the specific demands. So, future research should therefore overcome the terminological inequalities and develop a unified term to describe research on this type of training, such as the term FFT preferred by Dominski et al., which is independent of the CrossFit^®^ brand (Dominski et al., 2022).

## 5 Conclusion

The outcomes of this systematic review highlight specific research gaps and allow future research to be directed in this emerging field. In this way, the included studies show how CrossFit^®^ training in full extent affects the cardiovascular, endocrine, and inflammatory systems under practical conditions. For the first time, COVID-19 pandemic-induced effects on training behavior are also summarized. In addition, this review highlights the inconsistent use of the term CrossFit^®^ in sports science and recommends that further research implement more precise terminology. In this manner, physiological assessments enable coaches, athletes, and sports scientists to develop and implement effective training interventions and to recommend evidence-based practical applications. Overall, however, this review confirms that CrossFit^®^ is in many ways a sport with specific training practices exceeding the methods of HIFT or HIMT. However, limited information is available on the practical implementation. Therefore, further studies are needed to verify some of the assumptions.

## Data Availability

The original contributions presented in the study are included in the article/Supplementary Material, further inquiries can be directed to the corresponding author.

## Supplementary material

SUPPLEMENTARY FIGURE S1 Traffic light plot according to the Cochrane Collaboration’s risk of 537 bias assessment tool of included studies in Part A—Acute, short-term Physiological Responses.

SUPPLEMENTARY FIGURE S2 Traffic light plot according to the Cochrane Collaboration’s risk of bias assessment tool of included studies in Part B—Consequences of the COVID-19 Pandemic.
