# Supplementary figures and images for: Physiological effects of regular CrossFit® training and the impact of the COVID-19 pandemic—A systematic review
Source: Front Physiol. 2023 Apr 5;14:1146718. doi: 10.3389/fphys.2023.1146718 (PMC10113564; doi:10.3389/fphys.2023.1146718)

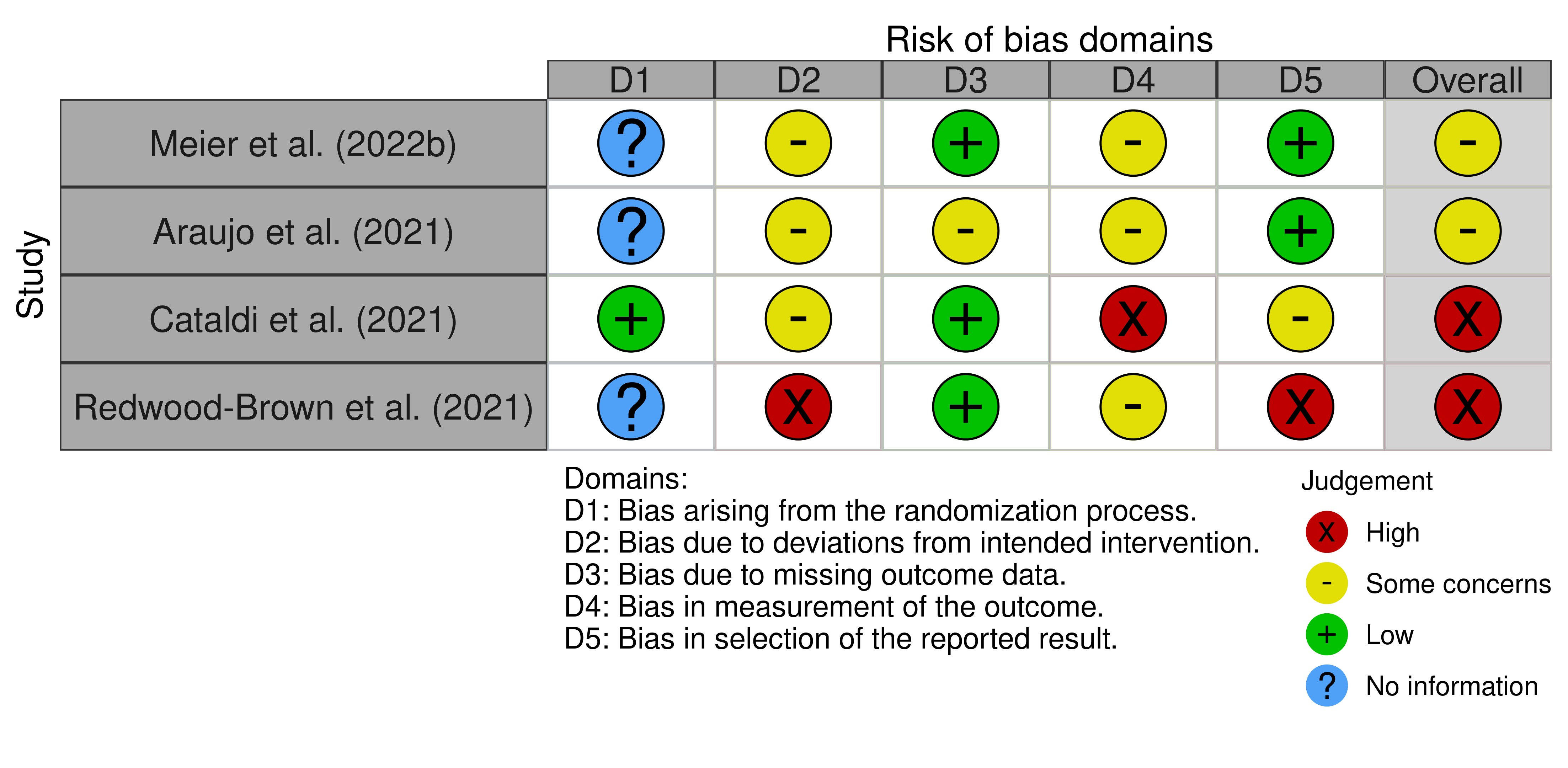

Supplement: Supplementary file 1 [file Image2.PNG]

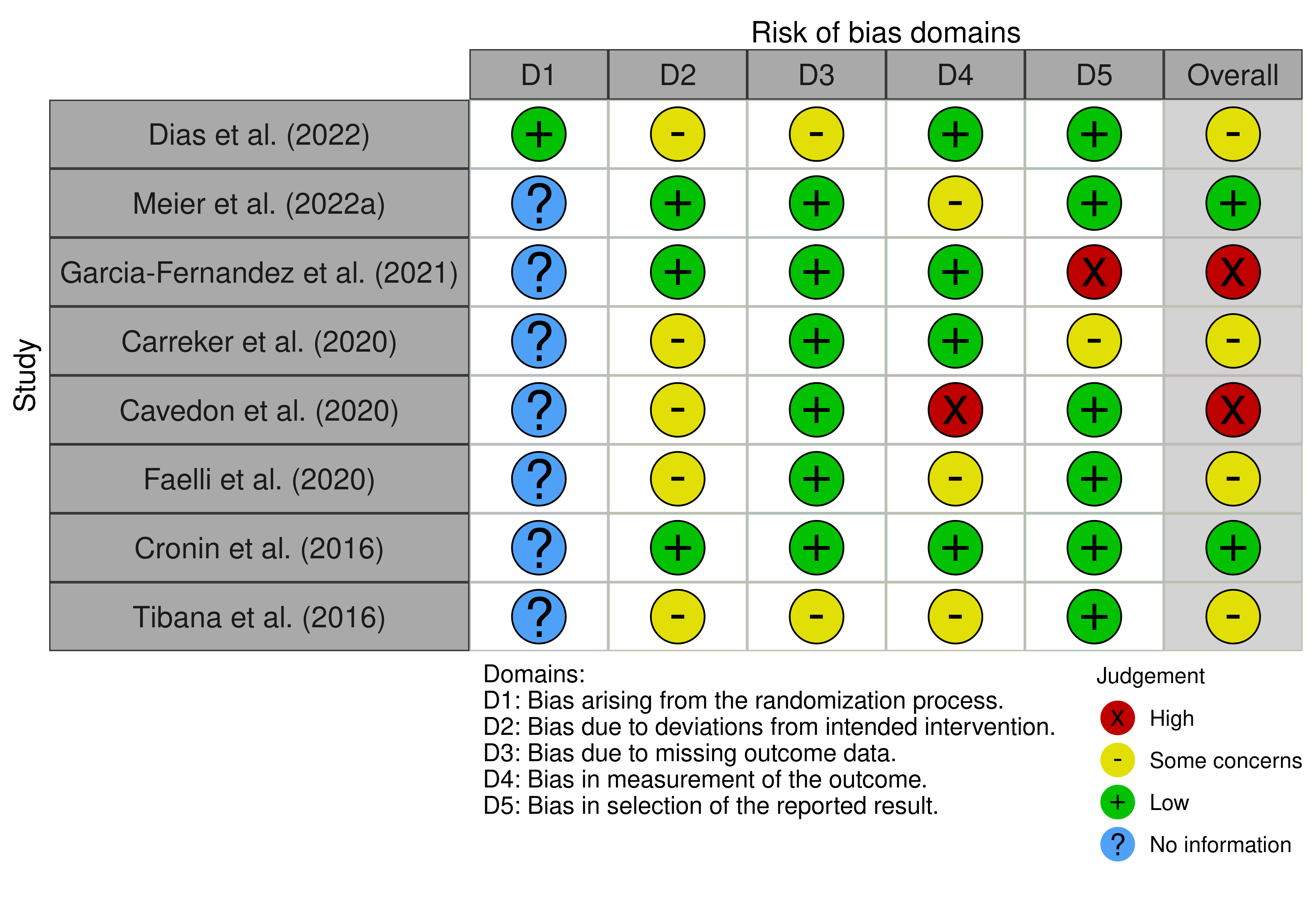

Supplement: Supplementary file 2 [file Image1.PNG]
